# Targeting Endothelial Cells with Multifunctional GaN/Fe Nanoparticles

**DOI:** 10.1186/s11671-017-2262-y

**Published:** 2017-08-10

**Authors:** Tudor Braniste, Ion Tiginyanu, Tibor Horvath, Simion Raevschi, Birgit Andrée, Serghei Cebotari, Erin C. Boyle, Axel Haverich, Andres Hilfiker

**Affiliations:** 1 National Center for Materials Study and Testing, Technical University of Moldova, Stefan cel Mare av. 168, MD-2004 Chisinau, Republic of Moldova; 2 Leibniz Research Laboratories for Biotechnology and Artificial Organs (LEBAO), Department of Cardiothoracic, Transplantation and Vascular Surgery, Hannover Medical School, Carl-Neuberg-Str. 1, D-30625 Hannover, Germany; 3 Department of Physics and Engineering, State University of Moldova, str. Alexei Mateevici 60, Chisinau, MD-2009 Republic of Moldova

**Keywords:** Nanoparticles, Gallium nitride, Endothelial cells, Encapsulation, Cell guiding

## Abstract

In this paper, we report on the interaction of multifunctional nanoparticles with living endothelial cells. The nanoparticles were synthesized using direct growth of gallium nitride on zinc oxide nanoparticles alloyed with iron oxide followed by core decomposition in hydrogen flow at high temperature. Using transmission electron microscopy, we demonstrate that porcine aortic endothelial cells take up GaN-based nanoparticles suspended in the growth medium. The nanoparticles are deposited in vesicles and the endothelial cells show no sign of cellular damage. Intracellular inert nanoparticles are used as guiding elements for controlled transportation or designed spatial distribution of cells in external magnetic fields.

## Background

In recent years, many efforts have been undertaken to combat cancer and related diseases using nanotechnology. One of the most common approaches is based on nanoparticles which can be exploited as drug carriers [1, 2]. This approach, however, has limitations related to the necessity of coating the nanoparticles with recognition ligands for drug adsorption and covalent binding, or caused by the need to encapsulate drugs within nanoparticles. An alternative therapeutic approach is to utilize nanoparticles for direct cell therapy, i.e., to target sites for the purpose of treating the disease biologically [3]. For example, endothelial cells loaded with magnetic nanoparticles could be guided to sites of arterial injury by means of an applied magnetic field. In addition to therapeutic applications, nanoparticle-assisted cell guiding can also be useful for in vitro cell separation and cellular coating of three-dimensional constructs [4]. In this paper, we demonstrate that endothelial cells take up GaN/Fe-based nanoparticles and that this phenomenon can be used to control the spatial distribution of cells in vitro.

## Methods

### Nanoparticle Synthesis

Thin layers of GaN were grown on ZnO nanoparticles alloyed with Fe_2_O_3_ by HVPE in two steps. Initially, the nucleation layer was deposited at 600 °C for 5 min. Subsequently, the temperature was increased to 800 °C and kept at this temperature for 10 min. The second temperature regime is necessary for ZnO core decomposition and improvement of GaN crystalline quality. The GaN growth has been described in detail by our group previously [5, 6]. In brief, we used metallic gallium, ammonia (NH_3_) gas, hydrogen chloride (HCl) gas, and hydrogen (H_2_) as carrier gases. In the process of GaN growth, the HCl, NH_3_, and H_2_ flow rates were 20, 600, and 3500 sccm, respectively.

### Cell Culture

Porcine aortic endothelial cells were isolated from aortas by gently scrapping of the endothelial cell layer with a scalpel. Cells were cultivated in a standard incubator at 37 °C with 5% CO_2_ in EGM™-2 (Endothelial Growth Factor Medium 2, Lonza). Cells splitting was performed with TrypLE™Select(1X) (Gibco®). For all experiments, cells between passage 3 and 8 were used. Cells were labeled with green fluorescence protein (GFP) by lentiviral transduction as described elsewhere [7].

### XTT Assay

The XTT assay was started 24 h after the medium change when new medium supplemented with nanoparticles was added. The culture medium was then replaced with fresh EGM2 medium with XTT reagent in a ratio of 2:1. The XTT reagent consists of 0.1 ml electron coupling reagent in 5 ml of XTT. After 4 h incubation at 37 °C with 5% CO_2_, the absorbance was measured on a Paradigm multi-mode plate reader.

### Cell Counting

After 2 days of incubation of cells with different concentrations of nanoparticles, cells were fixed in 4% paraformaldehyde for 10 min, washed with PBS, and stained with DAPI (1:7500 diluted in PBS) for 10 min. A random field of view was photographed from six independent wells with a high-resolution camera installed on a fluorescence microscope (Zeiss). Computer-assisted software DotCount v1.2 [8] was used for quantifying the relative number of cells in every well and compared to the control.

### Transmission Electron Microscopy

The transmission electron microscopy was performed after incubation of cells with nanoparticles for 1 day. After cells reached 50% confluence, culture medium was replaced with medium supplemented with 50 μg/ml GaN/Fe nanoparticles and cells were incubated for other 24 h. Cells were then washed with PBS, fixed in 2% glutaraldehyde and 2% formaldehyde at room temperature for 2 h, and then incubated overnight at 4 °C. The samples were washed in 0.1 M sodium cacodylate and post-fixed in 1% OsO_4_ in 0.1 M sodium cacodylate for 1 h. After fixation, the samples were dehydrated in a graded acetone series and embedded in EPON. Polymerization was performed for 2 days at 60 °C. Thin sections of ~50 nm thick were collected on formvar-coated copper slot grids and stained with 4% uranyl acetate and lead citrate. Cellular sections were investigated in detail using a transmission electron microscope FEI Tecnai 20 at an acceleration voltage of 200 kV.

## Results and discussion

**Fig. 1:**
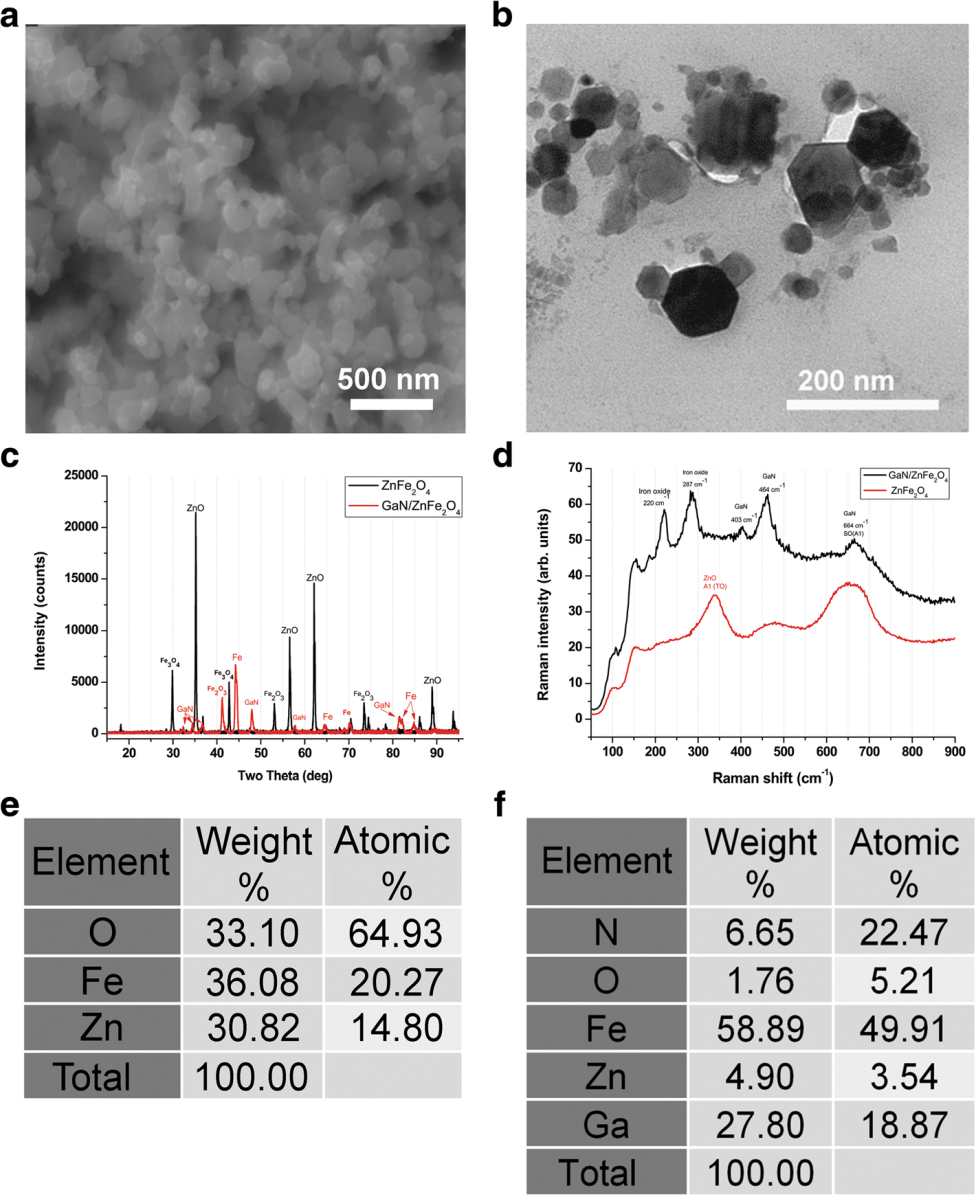
Analysis of nanoparticles. **a** SEM picture of GaN nanoparticles grown on sacrificial nanoparticles of ZnO alloyed with Fe_2_O_3_. **b** TEM image of the resultant GaN/Fe nanoparticles. **c** XRD pattern of initial ZnFe_2_O_4_ nanoparticles and resultant GaN/ZnFe_2_O_4_ nanoparticles. **d** Raman spectra of the initial and resultant nanoparticles after GaN growth. **e** EDX analysis of ZnO alloyed with Fe_2_O_3_ nanoparticles. **f** EDX analysis of resulted nanoparticles after the growth of the GaN layer

Multifunctional magnetic nanoparticles have been fabricated by growing a GaN layer on sacrificial nanoparticles of ZnO alloyed with Fe_2_O_3_. After growth of the GaN layer using hydride vapor phase epitaxy (HVPE), the ZnO core is decomposed. The resulted chemically stable nanoparticles consist mainly of a GaN shell with magnetic properties attributable to the diffusion of iron atoms in the deposited GaN as well as to the presence of Fe atoms in the thin film of ZnO alloyed with Fe_2_O_3_ on the inner surface of the GaN shell. These nanoparticles were investigated using electron microscopy. After the HVPE growth process of GaN, the single crystalline nanoparticles with transverse sizes ranging from 20 to 100 nm remain spatially separated (Fig. 1). The results of X-ray diffraction and Raman spectroscopy characterization (Fig. 1c, d) of the nanoparticles before and after GaN growth demonstrate the decomposition of the ZnO core and the formation of GaN nanoparticles. Chemical analyses of the nanoparticles performed by using energy-dispersive X-Ray analysis (EDX) confirm the growth of the GaN layer and the decomposition of the ZnO core (Fig. 1e, f). Note that the resultant material display a relatively high (approximately 50%) concentration of Fe comparing to the initial nanoparticles.

**Fig. 2:**
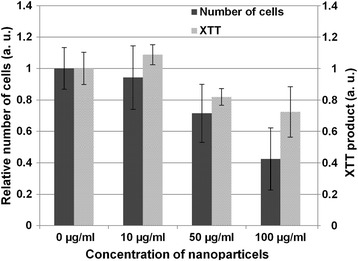
Impact of nanoparticles on cell viability. Concentration-dependent XTT reduction measured after 1 day of cells being incubated with different concentrations of nanoparticles. The number of cells counted at the end of the XTT assay is expressed relative to untreated cells. Values are expressed as means ± standard deviation of two independent experiments with six replicates

GaN/Fe-based nanoparticles were incubated with primary porcine aortic endothelial cells. As was previously shown, GaN nanoparticles are tolerated by the endothelial cells in concentrations less than 100 μg/ml [5]. During the incubation process, endothelial cells take up the majority of the nanoparticles in the surrounding culture medium while maintaining cell migration and proliferation. Nevertheless, we noticed some decrease in the number of viable cells with an increase in the concentration of nanoparticles in the culture media. This tendency is confirmed by the results of the XTT assay presented in Fig. 2.

**Fig. 3:**
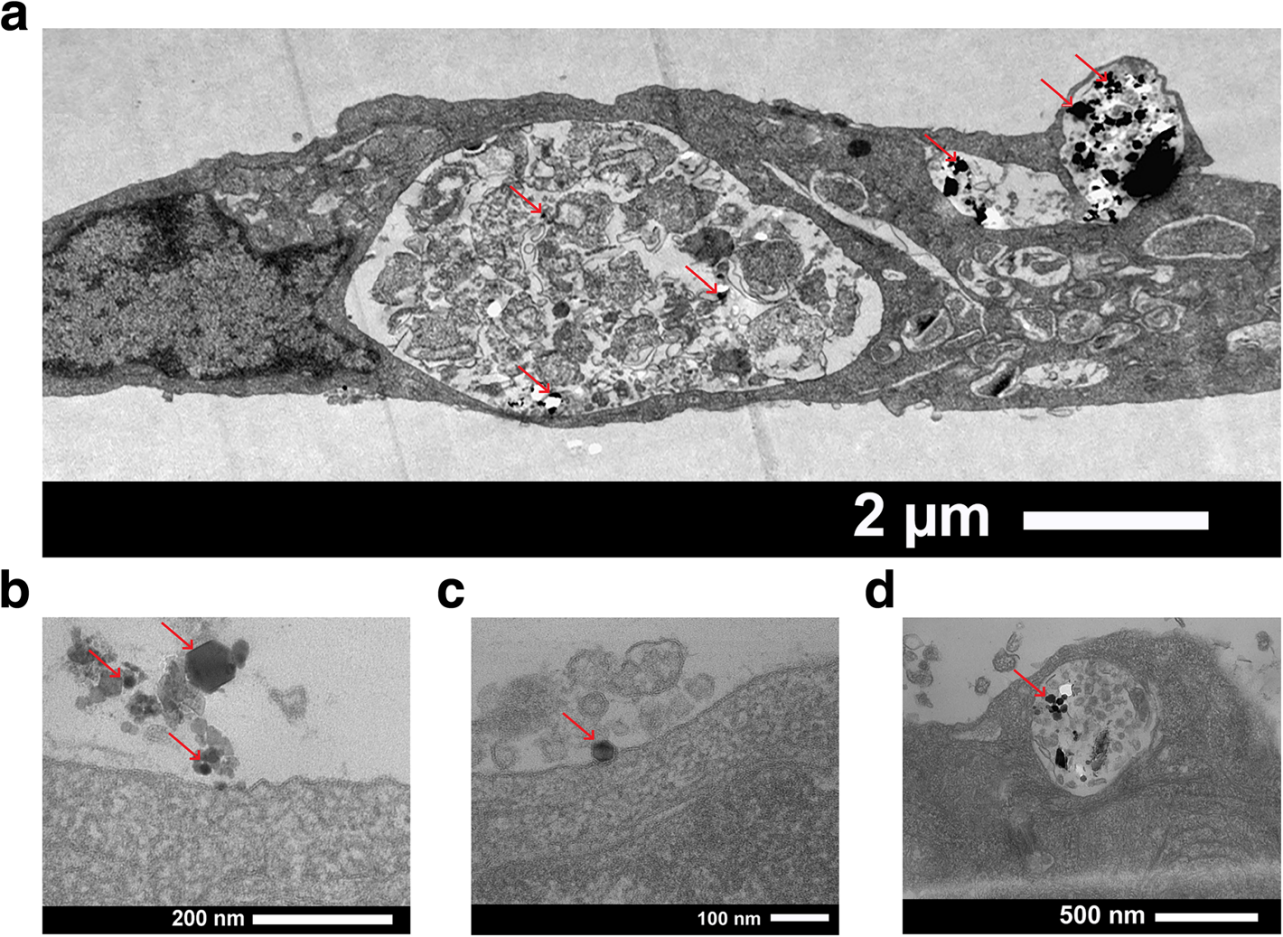
TEM pictures taken from a single endothelial cell incubated with GaN/Fe nanoparticles. **a** Nanoparticles distribution within cellular vesicles. **b**–**d** The uptake process of nanoparticles into vesicles. *Red arrows* indicate nanoparticles that appear darker in TEM due to high atomic density compared to biological media

To understand how GaN/Fe nanoparticles interact with cells and to identify their localization within cells, we performed thorough morphological analysis using transmission electron microscopy (TEM). After incubation of porcine aortic endothelial cells with 50 μg/ml nanoparticles for 1 day, the nanoparticles proved to be localized in vesicles inside the cells (Fig. 3a). No nanoparticles were found in the cytoplasm or in the cell nucleus. The uptake process of nanoparticles is presented in Fig. 3b–d. Most of nanoparticles are taken up by cells through one of the classical uptake pathways, namely through micropinocytosis, clathrin-mediated endocytosis, or caveolin-mediated endocytosis [9]. The internalization process depends on the cell type and local cellular environment as well as on the physiochemical properties of the particle itself (e.g., size, shape, surface charge). In the case of endothelial cells, caveolin-mediated endocytosis was reported to have a higher influence on nanoparticle uptake than other mechanisms due to the abundance of caveolin in this cell type [10, 11].

**Fig. 4:**
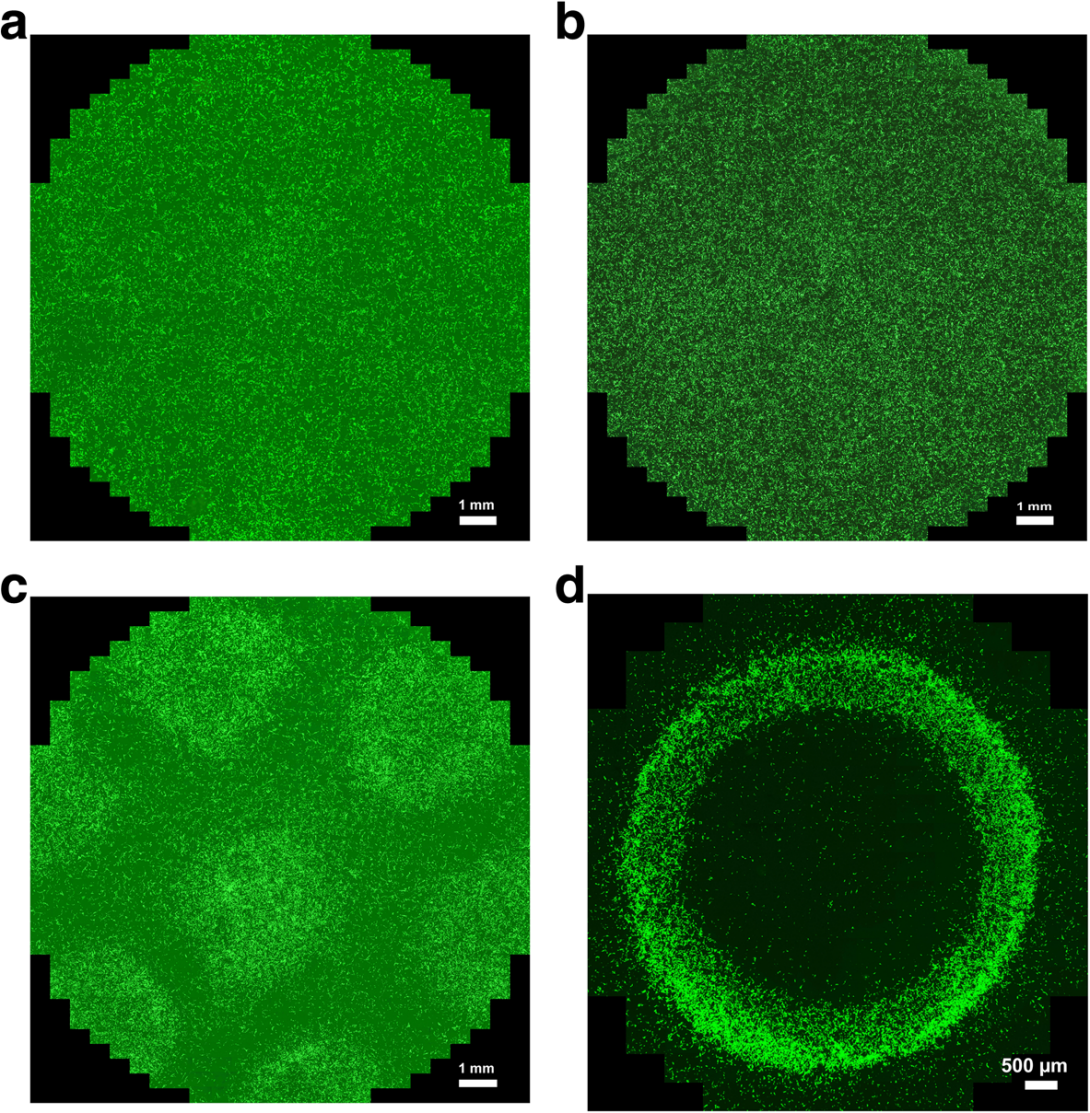
Guiding of nanoparticle-laden endothelial cells using a magnetic field. The control group shows the spatial distribution of **a** endothelial cells targeted with nanoparticles and incubated in the absence of magnetic field and **b** nanoparticle-free endothelial cells incubated in magnetic field. **c**, **d** The distribution of endothelial cells targeted with nanoparticles after 1 day of incubation in a magnetic field

Due to the aforementioned incorporation of a high amount of Fe, the resultant nanoparticles exhibit ferromagnetism, along with piezoelectricity inherent to GaN semiconductor material [12, 13]. These two fundamental properties can be used for remote activation of some processes in the nanoparticles and/or their controlled guiding and spatial distribution in relevant media. Piezoelectric properties can be used to induce electrical polarization in GaN nanoparticles by, for example, an applied ultrasound field. In this way, one can transmit electrical signals to the cells to activate or inhibit specific cellular processes. As to the magnetic properties conferred by the Fe content, they enable one to reach dynamic visualization and control of the spatial position of cells. To experimentally demonstrate the latter possibility, endothelial cells were incubated in EGM™-2 medium supplemented with 50 μg/ml of GaN/Fe nanoparticles for 3 days (until 70–80% cell confluence). Subsequently, the cells were detached from the surface and re-suspended in EGM™-2. Note that the detachment of cells with TrypLE™ Select and centrifugation have neither affected the cell viability nor resulted in the release of nanoparticles from the cells (data not shown). Immediately after seeding, the cells were incubated in a standard incubator at 37 °C under 5% CO_2_, where the culture plate was placed on permanent magnets. Figure 4 shows the distribution of nanoparticle-laden endothelial cells in the presence and absence of a magnetic field. Figure 4a depicts nanoparticle-laden cells incubated in the absence of a magnetic field, while in Fig. 4b, endothelial cells without nanoparticles are incubated in a magnetic field. These pictures show a random distribution of cells in both cases. Incubation of nanoparticle-laden cells in a magnetic field gradient leads to a pre-designed distribution of cells in certain areas, in accordance with the magnetic field map. Figure 4c depicts cells in the culture plate after 1 day of incubation in the magnetic field generated by seven rare earth neodymium circular magnets with a diameter of 5 mm and a thickness of 1 mm. Figure 4d illustrates cell distribution after incubation in the magnetic field generated by a single ring-shaped magnet with a diameter of 7 mm and a thickness of 1 mm. In both cases, the magnets were placed below the culture plate.

## Conclusions

We have demonstrated for the first time that the GaN/Fe-based nanoparticles exhibiting magnetic properties are taken up by endothelial cells and stored within vesicles. The GaN/Fe nanoparticle-laden endothelial cells can be guided in a controlled fashion using applied magnetic fields. These results open new possibilities for engineering three-dimensional tissues in vitro or for targeting cells in vivo to sites of tissue injury. Along with this, the presence in the cells of GaN nanoparticles with inherent piezoelectric properties paves the way for remote electrical stimulation of cellular biological processes. This promising approach is under investigation in our laboratories.

## Abbreviations

EDX: Energy dispersive X-ray analysis
EGM™-2: Endothelial Growth Factor Medium
Fe: Iron
Fe_2_O_3_: Iron oxide (III)
GaN: Gallium nitride
GFP: Green fluorescence protein
H_2_: Hydrogen
HCl: Hydrogen chloride
NH_3_: Ammonia
OsO_4_: Osmium tetroxide
PBS: Phosphate buffered saline
SEM: Scanning electron microscopy
TEM: Transmission electron microscopy
XRD: X-ray diffraction
ZnO: Zinc oxide
